# The independent association between 25 (OH) vitamin D deficiency, HOMA-IR, and lipid profile with APOE genotyping in obese cases with and without T2DM

**DOI:** 10.1186/s13098-024-01427-4

**Published:** 2024-08-13

**Authors:** Nagla Usama, Amr El-Sayed, Mohamed Gamal, Salma Mekheimer, Khaled Elhadidy, Mohamed Awadein, Mahmoud Farid

**Affiliations:** 1 Medical Laboratory Technology Department, Faculty of Applied Health Science Technology, Misr University for Science and Technology, Cairo, Egypt; 2 Biotechnology and Life Sciences Department, Faculty of Postgraduate Studies for Advanced Sciences, Beni-Suef University, Beni-Suef, Egypt; 3 Internal Medicine Gastroenterology and Hepatology, Faculty of Medicine, Ain Shams University, Cairo, Egypt; 4 Internal Medicine Department, Faculty of Medicine, Beni-Suef University, Beni-Suef, Egypt; 5 Internal Medicine Department, Faculty of Medicine, Misr University for Science and Technology, Cairo, Egypt

## Abstract

**Introduction:**

Vitamin D deficiency, insulin resistance, dyslipidemia, and APOE genotyping are implicated in the pathogenesis of obesity and type 2 diabetes mellitus (T2DM). We wanted to find out if there was a link between a lack of 25(OH) vitamin D, HOMA-IR, and lipids and APOE genotyping in obese people with and without T2DM.

**Methods:**

We divided 300 Egyptians of both sexes into three groups in a case-control study: 100 obese cases with a body mass index of more than 30, 100 obese cases diagnosed with T2DM, and 100 controls with a body mass index of less than 30. Levels of 25 (OH) vitamin D, fasting blood sugar (FBS), HbA1C, fasting insulin, HOMA-IR, and lipid profile parameters were measured, and APOE genotypes were assessed using Applied BiosystemsTM TaqMan^®^ SNP Genotyping Assays.

**Results:**

Higher levels of FBS, fasting insulin, HOMA-IR, and dyslipidemia were found in obese people with and without T2DM compared to the control group (*p* < 0.05). Lower levels of 25(OH) vitamin D were also found. Insulin resistance and lipid profile parameters, particularly in obese cases with T2DM, inversely correlate with vitamin D deficiency. The APOE genotyping analysis revealed strong links between vitamin D levels and certain APOE genotypes. Independent of metabolic parameters, higher vitamin D levels were associated with lower odds of E3/E4 and E4/E4 genotypes among obese cases with T2DM.

**Conclusion:**

This study highlights the independent role of vitamin D deficiency in modulating APOE genotypes in obese T2DM individuals. The findings suggest potential implications for personalized interventions targeting vitamin D status to mitigate genetic predispositions to metabolic disorders such as obesity and T2DM.

## Introduction

Type 2 diabetes mellitus (T2DM), a metabolic condition characterized by chronic hyperglycemia and impaired protein, lipid, and carbohydrate metabolism, can result from either inadequate insulin production or a decreased sensitivity to its metabolic effects. Obesity and type 2 diabetes mellitus (T2DM) are both on the rise around the world. Obesity and insulin resistance have a common link, and obesity is a pathophysiologic cause of type 2 diabetes mellitus. Insulin resistance is the damage to the physiological response of insulin-stimulated target tissues, most commonly the liver, muscles, and adipose tissue. Impaired glucose elimination due to insulin resistance causes hyperinsulinemia and an increase in beta-cell insulin production [1–3].

Obesity is associated with abnormalities in adipose tissue (AT) function and the buildup of body fat, which in turn increases the risk of metabolic syndrome, insulin resistance, type 2 diabetes, and cardiovascular disease. Obesity is associated with low plasma 25(OH)D3 levels (50 nmol/L), which is an intriguing finding. People who are overweight often have low plasma vitamin D levels, which can lead to metabolic disorders such as insulin resistance and type 2 diabetes [4]. It was suggested that insulin resistance and vitamin D insufficiency go hand in hand and that hypovitaminosis D may play a part in the onset of type 2 diabetes mellitus in those who are overweight. It seems that hypovitaminosis D contributes to the development of many metabolic disorders in both adults and children. Apart from the well-known calciotropic benefits of vitamin D, multiple clinical trials have demonstrated that supplementing with vitamin D in individuals with type 2 diabetes and metabolic syndrome improves insulin sensitivity, glycated hemoglobin (HbA1c), and lipid profiles [5].

The apolipoprotein E (APOE) gene is located on the long arm of chromosome 19 at position q13.32. This gene encodes a 299-amino-acid glycoprotein. It is a cholesterol transporter and plays a role in the metabolism, transport, and digestion of several different lipoproteins by acting as a high-affinity ligand for multiple hepatic lipoprotein receptors, including LDL-R and LDL-related protein (LRP1) [6, 7]. The APOE gene has three major alleles: ε2, ε3, and ε4, which are derived from two single-nucleotide polymorphisms (SNPs), namely, rs429358 (T > C) and rs7412 (C > T). The three alleles form six different genotypes: ϵ2/ϵ2, ϵ3/ϵ3, ϵ4/ϵ4, ϵ2/ϵ3, ϵ2/ϵ4, and ϵ3/ϵ4. Furthermore, these genotypes code for three distinct protein isoforms: APOE2 (ϵ2/ϵ2, ϵ2/ϵ3; Cys112/Cys158), APOE3 (ϵ2/ϵ4, ϵ3/ϵ3; Cys112/Arg158), and APOE4 (ϵ3/ϵ4, ϵ4/ϵ4; Arg112/Arg158) [8].

The ε3 is the most common allele, with a prevalence of 70–80%. The ε2 is present in 5–10% and the ε4 is present in 10–15% of individuals [9]. Different APOE isoforms are associated with considerable variance in lipid profiles [10]. Previous research has revealed that different APOE alleles can affect lipid clearance and metabolism. There is evidence that APOE 2 allele carriers have higher APOE plasma levels, lower LDL cholesterol (LDL-C) plasma levels, and a reduced risk of coronary artery disease (CAD), while APOE4 allele carriers have lower APOE plasma levels, higher total cholesterol (TC), LDL cholesterol (LDL-C), and very low density lipoprotein cholesterol (VLDL-C) plasma levels, and a higher risk of CAD when compared to APOE3 homozygotes [11].

Researchers have studied the relationship between Apo E polymorphisms and T2DM, obesity, and metabolic syndromes [11–13]. Obesity and type 2 diabetes are the most frequent and widespread diseases in the world. Obesity intensifies the effects of genetic susceptibility and environmental factors on diabetes by sharing strong genetic and environmental characteristics in their development [14]. This study is designed to investigate the independent association between 25 (OH) vitamin D deficiency, HOMA-IR, and lipid profile with APOE genotyping in obese cases with and without T2DM.

## Materials and methods

We designed a case-control study to assess 300 Egyptian cases, divided into 100 obese cases, consisting of 31 males (31%) and 69 females (69%). Their body mass index is more than 30. The 100 obese cases diagnosed with T2DM consisted of 38 males (38%) and 62 females (62%); their body mass index was more than 30; and their serum 25-hydroxyvitamin D level was less than 20 ng/dl. and 100 cases as controls consisted of 29 males (29%) and 71 females (71%); their body mass index was less than 30. and their serum 25-hydroxyvitamin D level was more than 20 ng/dl.

The Souad Kafafi University Hospital’s diabetic clinic served as the recruitment site for all studied cases. Each case provided written informed consent, and the MUST University Ethics Committee (FWA00025577) approved the research protocol.

Tools for data collection: The assessment sheet consists of demographic data (age, sex, and BMI) and biochemical analysis (25-hydroxyvitamin D, fasting blood sugar, HbA1c, fasting insulin, HOMA-IR, and lipid profile). The real-time polymerase chain reaction (RT-PCR) technique detects Apo E gene polymorphisms (rs7412 and rs429358) through a genotyping assay that includes DNA extraction.

### Biochemical analyses of blood

Overnight fasting venous blood samples were collected from all cases in three different types of anticoagulant tubes using standardized protocol and equipment: an EDTA-containing tube used for DNA extraction, APOE gene SNP detection, and the measurement of glycated hemoglobin; a sodium fluoride-containing tube used for measuring fasting blood sugar; and a tube containing no anticoagulant used for lipid profile and fasting insulin. We measured fasting blood sugar and lipid profile (total cholesterol, triglyceride, and HDL-C) in serum using an XL180 fully automatic clinical chemistry analyzer (Erba Mannheim), and calculated LDL cholesterol using the Friedewald equation. the turbidimetric method on semi-automated chemistry analyzers using a spectrum diagnostic kit to quantitatively determine HbA1c. The PerkinElmer ELISA kit determined 25-hydroxyvitamin D in human serum. The catalog number is 10,501. The criteria to characterize 25-hydroxyvitamin D deficiency are based on Trimboli, F. et al.

The Chemux Bioscience, Inc. ELISA kit determined the insulin concentrations in human serum. Catalog number: 1080. The main HOMA model uses a simplistic, nonlinear mathematical equation to describe the equilibrium between glucose and insulin. The following formulas form the basis of its calculation [15]:

HOMA1-IR = (fasting blood sugar (mg/L) × fasting insulin (mU/L))/405.

Molecular and genotyping assay:

DNA extraction: Using Thermo Scientific Gene JET Whole Blood Genomic DNA Purification Mini Kit #K0781, #K0782.

Applied BiosystemsTM TaqMan^®^ SNP Genotyping Assays performed genotyping of APOE gene polymorphisms (rs7412 and rs429358) using TaqMan^®^ 5nuclease chemistry for amplifying and detecting specific polymorphisms in purified genomic DNA samples.

### APOE genes SNPs

SNP ID: (rs7412) Location: Chr.19:44908822 on Build GRCh38.

Context Sequence [VIC/FAM] CCGCGATGCCGATGACCTGCAGAAG[C/T] GCCTGGCAGTGTACCAGGCCGGGGC.

SNP ID: (rs429358) Location: Chr.19:44908684 on Build GRCh38.

Context Sequence [VIC/FAM] GCTGGGCGCGGACATGGAGGACGTG[C/T] GCGGCCGCCTGGTGCAGTACCGCGG.

Each TaqMan^®^ SNP Genotyping Assay has two TaqMan^®^ minor groove binder (MGB) probes with nonfluorescent quenchers (NFQ): one VIC^®^-labeled probe to find the Allele 1 sequence and one FAM^®^-labeled probe to find the Allele 2 sequence. The forward and reverse primers are specific to the polymorphic sequence of interest.

The PCR conditions were changed in a 10 µl mixture that had 7 µl of 2X TaqMan^®^ Master Mix, 0.3 µL of 20X assay working stock, and 2.70 µL of DNA sample (conc. 20 ng). PCR began with a pre-PCR holding stage at 95 °C (30s), then 40 cycles at 95 °C (10s) and 60 °C (60s), followed by a final extension at 60 °C (30s).

### Statistical analysis

We collected and entered data into the computer using the SPSS (Statistical Package for Social Science) program for statistical analysis (version 26, Inc., Chicago, IL). We conducted two types of statistics: descriptive statistics, which presented quantitative data as mean, and SD, and qualitative data as frequency and percentage. The genotype frequencies of the two polymorphisms were tested for Hardy-Weinberg equilibrium (HWE) using the goodness-of-fit (chi-square) test. We used a student t-test to compare the mean and SD of two sets of quantitatively normally distributed data. A one-way analysis of variance (ANOVA) test was applied, and the significant differences among values were analyzed with Duncan’s new multiple range tests at *p* < 0.05. We performed the chi-square test to examine the degree of correlation between the various qualitative factors. We used logistic regression to estimate odds ratios (OR) and 95% confidence intervals (CI). The pearson correlation matrix was achieved using the R programming Language version 4.3.3 with RStudio open-source version 04.0-735.

## Results

Table 1 summarizes the main demographic and biochemical characteristics of the study population as follows: This study included 100 obese cases, consisting of 31 males (31%) and 69 females (69%). The mean value (µ ± SD) of their age is 43.2 ± 4.6, and the mean value (µ ± SD) of their body mass index is 35.6 ± 4.7. 100 obese cases diagnosed with T2DM consisted of 38 males (38%) and 62 females (62%); the mean value (µ ± SD) of their age was 42.8 ± 5.4, and the mean value (µ ± SD) of their body mass index was 32.85 ± 3.4. 100 cases as controls consisted of 29 males (29%) and 71 females (71%); the mean value (µ ± SD) of their age was 42.7 ± 3.7, and the mean value (µ ± SD) of their body mass index was 24.6 ± 2.2. All studied groups showed no statistically significant difference with age or sex (P-value > 0.05). and had significantly difference with MBI (P-value < 0.05).

Concerning the 25 (OH) vitamin D levels. The results indicated that the control group had insufficient levels of 25-OH vitamin D, with the mean value (µ ± SD) of 25.2 ± 3.1. Notable discrepancies were observed when comparing these outcomes among obese individuals with and without Type 2 Diabetes Mellitus. (P-value < 0.001).

Furthermore, FBS, HbA1C, Fasting insulin and HOMA-IR levels were significantly higher in both obese and T2DM obese cases compared to the control group. The lipid profile results showed significant elevations in total cholesterol, triglycerides, LDL-C levels, and TG/HDL-C ratio in obese individuals with and without T2DM compared to the control group. However, among obese individuals with and without type 2 diabetes mellitus, there was no significant difference in TC, TG, LDL-C levels, or TG/HDL-C ratio levels between obese individuals with type 2 diabetes and the control group (*p* > 0.05).

**Table 1:** Demographic and biochemical data and for all cases

Parameter	Mean (µ ± SD), *n*%			*P*-value		
	Control group. *N* = 100	Obese cases *N* = 100	Obese cases with T2DM *N* = 100	Control Vs Obese cases	Control Vs Obese cases with T2DM	Obese cases Vs Obese cases with T2DM
Sex	Male (29) 29% Female (71) 71%	Male (31) 31% Female (69) 69%	Male (38) 38% Female (62) 62%			
Age	42.7 ± 3.7	43.2 ± 4.6	42.8 ± 5.4	0.397	0.813	0.626
BMI	24.6 ± 2.2	35.6 ± 4.7	32.85 ± 3.4	< 0.001	< 0.001	< 0.001
25(OH) vitamin D	25.2 ± 3.1	10.1 ± 4.9	10.2 ± 4.3	< 0.001	< 0.001	0.868
FBS (mg/dl)	85.7 ± 9.3	105.6 ± 20.2	131.3 ± 40.6	< 0.001	< 0.001	< 0.001
HbA1C %	4.7 ± 0.42	5.5 ± 0.7	7.8 ± 1.2	< 0.001	< 0.001	< 0.001
Fasting insulin mU/L	5.4 ± 3.4	7 ± 4.8	8.2 ± 3.6	0.007	< 0.001	0.046
HOMA-IR	1.1 ± 0.7	1.8 ± 1.3	2.6 ± 1.74	< 0.001	< 0.001	0.002
TC (mg/dl)	152.5 ± 28.2	183.8 ± 38.5	181.2 ± 40.1	< 0.001	< 0.001	0.632
TG (mg/dl)	129 ± 33.7	164.1 ± 62.7	148.7 ± 51	< 0.001	0.002	0.059
HDL-C (mg/dl)	55.9 ± 10.2	55.5 ± 8.6	50.1 ± 9.1	0.757	< 0.001	< 0.001
LDL-C (mg/dl)	70. ± 25.337	95.5 ± 33	101.3 ± 37.6	< 0.001	< 0.001	0.248
TG/HDL-C ratio	1.96 ± 0.721	3.05 ± 1.3	3.1 ± 1.3	< 0.001	< 0.001	0.729

**Fig. 1:**
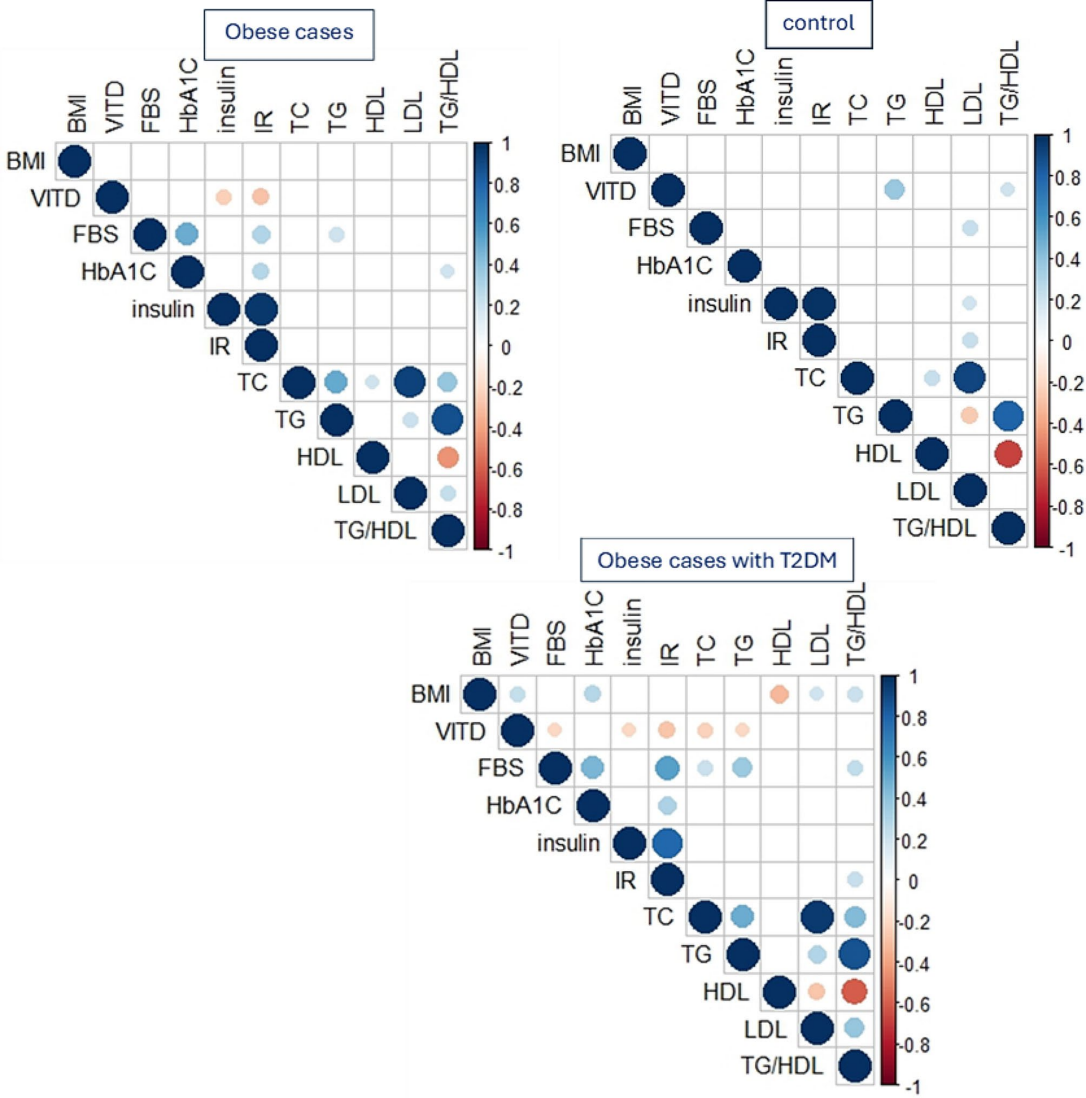
Illustrated the Pearson correlation matrix for demographic and biochemical parameters between control group, Obese cases, and obese cases with T2DM. Statistically significant correlations were only shown

Pearson correlation for demographic and biochemical data among all cases illustrated in Fig. (1): The results revealed a significant negative correlation between low levels of 25 (OH) vitamin D and fasting insulin level as well as HOMA-IR, with correlation coefficients of -0.234 and − 0.288 respectively. The p-value was less than 0.05 in obese cases. There was a significant inverse correlation with FBS, fasting insulin level, HOMA-IR, TC, and TG with correlation coefficients of -0.201, -0.201, -0.275, -0.230, and − 0.202 correspondingly. The p-value was less than 0.05 in obese individuals with T2DM.

### Genotyping and allelic frequencies

The Hardy-Weinberg equilibrium test was used to compare the results of observed values with expected values for the three SNP loci genotypes APOE (rs42938 & rs7412) among all studied cases. The results showed that the difference in the rs429358 site was not statistically significant among obese cases (*P* > 0.05) and statistically significant difference among T2DM cases and control group, and the rs7412 sites had statistically significant differences among obese cases and T2DM cases but it had not among control group. (*P* > 0.05). Table 2 illustrates the distribution of APOE genotyping and alleles frequencies in all studied groups. For APOE, The APOE Ɛ3/Ɛ4 was the most frequent genotype between the studied cases and control group, T2DM obese cases represented (39%), obese cases represented (31%), and control group represented (50%). Ɛ3 allele was the most frequent allele in obese cases (43%) and control group (39.5%), while Ɛ4 allele was the most frequent allele in T2DM obese cases (43.5%).

**Table 2:** The distribution of APOE genotyping in all studied cases

APOE genotyping & alleles	Control group. *N* = 100	Obese cases *N* = 100	obese cases with T2DM *N* = 100	P- value		
				Control Vs obese cases	Control Vs obese cases withT2DM	Obese cases without and with T2DM
E3/E3 (ref)	19 (19%)	21 (21%)	13 (13%)	0.055	< 0.001	0.005
E2/E2	0	3 (3%)	7 (7%)			
E2/E3	15 (15%)	26 (26%)	10 (10%)			
E2/E4	20 (20%)	11 (11%)	22 (22%)			
E3/E4	46 (46%)	39 (39%)	31 (31%)			
E4/E4	0	0	17 (17%)			
E3 (ref)	99 (45.5%)	79 (39.5%)	67 (33.5%)	< 0.001	0.005	< 0.001
E2	35 (17.5%)	71 (35.5%)	46 (23%)			
E4	66 (33%)	50 (25%)	87 (43.5%)			

Table 3 illustrates the associations of APOE gene polymorphisms and the risk of obesity and T2DM compared to the control group using univariate analysis. Ɛ4/Ɛ4 is associated with obese cases with T2DM compared with both obese cases without T2DM (OR = 2.308, 95% CI = 1.533–3.475, P-value < 0.001) and the control group (OR = 0.433, 95% CI = 0.288–0.652, P-value < 0.001). Ɛ2/Ɛ2 is associated with obese cases with and without T2DM compared with the control group (OR = 1.412, 95% CI = 1.092–1.825, P-value = 0.010) & (OR = 1.231, 95% CI = 0.973–1.557, P-value = 0.048 respectively. Additionally, the E4 allele is associated with T2DM obese cases compared with obese cases without T2DM (OR = 2.052, 95% CI = 1.274–3.304, P-value = 0.003). and with control group (OR = 1.948, 95% CI = 1.248–3.041, P-value = 0.003). Furthermore, E2 allele is associated with obese cases with and without T2DM compared to control group.

**Table 3:** Associations of APOE genes polymorphisms and the risk of obesity with and without T2DM

APOE genotyping & allele	Obese cases vs. control		obese cases with T2DM vs. control		Obese cases without and with T2DM	
	OR (95%CI)	*p*-value	OR (95%CI)	*p*-value	OR (95%CI)	*p*-value
E3/E3 (ref)	-	-	-	-	-	-
E2/E2	1.231 (0.973–1.557)	0.048	1.412 (1.092–1.825)	0.010	0.293 (0.064–1.348)	0.104
E2/E3	2.533 (0.980–6.547)	0.053	0.974 (0.335–2.831)	0.962	1.779 0.645–4.907	0.264
E2/E4	0.804 (0.290–2.228)	0.674	1.608 (0.635–4.074)	0.316	0.622 0.245–1.576	0.316
E3/E4	0.985 (0.425–2.281)	0.972	0.985 (0.425–2.281)	0.972	1.015 0.438–2.351	0.972
E4/E4	-	-	2.308 (1.533–3.475)	< 0.001	0.433 (0.288–0.652)	< 0.001
E3 (ref)	-	-	-	-	-	-
E2	2.542 (1.540–4.197)	< 0.001	1.942 (1.134–3.326)	0.015	0.764 (0.466–1.251)	0.284
E4	0.949 (0.592–1.522)	0.829	1.948 (1.248–3.041)	0.003	2.052 (1.274–3.304)	0.003

Figure 1 illustrated the results obtained from one way ANOVA test conducted to compare the values of biochemical parameter between APOE genotypes among obese cases and obese cases with T2DM, and the significant differences among values were analyzed with Duncan’s new multiple range tests at *p* < 0.05. The low levels of 25 (OH) vitamin D were significantly difference in APOE genotypes among obese cases whereas E2/E4 and E3/E4 had the lowest among obese cases, and E2/E4, E3/E4, and E4/E4 had the lowest among obese cases with T2DM.

Multivariate logistic regression analysis was performed to evaluate 25 (OH) vitamin D deficiency as independent risk variables with the APOE genotyping (dependent variable) among obese cases (Table 4) and obese case with T2DM (Table 5).

**Table 4:** Logistic regression analysis among obese cases

	E2/E2		E2/E3		E3/E4		E2/E4	
	B	OR 95%CI	B	OR 95%CI	B	OR 95%CI	B	OR 95%CI
Model 1	0.08	1.08 (0.79–1.492) **p-value** 0.60	-0.1	0.90 (0.78–1.04) **p-value** 0.185	-0.50	0.60 (0.49–0.74) **p-value** <0.001	-0.31	0.735 (0.59–0.91) **p-value** 0.006
Model 2	0.018	1.018 (0.71–1.44) **p-value** 0.92	-0.15	0.85 (0.72–1.01) **p-value** 0.069	-0.59	0.55 (0.43–0.69) **p-value** < 0.001	-0.33	0.71 (0.56–0.9) **p-value** 0.005
Model 3	-0.037	0.96 (0.64–1.438) **p-value** 0.856	-0.17	0.83 (0.697–1.00) **p-value** 0.062	-0.80	0.44 (0.33–0.60) **p-value** < 0.001	-0.43	0.64 (0.47–0.88) **p-value** 0.006

**Table 5:** Logistic regression analysis among obese cases with T2DM

	E2/E2		E2/E3		E3/E4		E2/E4		E4/E4	
	B	OR 95%CI	B	OR 95%CI	B	OR 95%CI	B	OR 95%CI	B	OR 95%CI
Model 1	0.14	1.15 (0.92–1.43) **P-value** 0.196	0.097	1.10 (0.90–1.34) **P-value** 0.346	-0.58	0.55 (0.42–0.73) **P-value** < 0.001	-0.33	0.71 (0.55–0.92) **P-value** 0.011	-0.42	0.65 (0.49–0.87) **P-value** 0.004
Model 2	0.10	1.107 (0.866–1.417) **P-value** 0.41	0.147	1.158 (0.91–1.46) **P-value** 0.22	-0.57	0.56 (0.419–0.75) **P-value** < 0.001	-0.32	0.72 (0.55–0.95) **P-value** 0.02	-0.41	0.66 (0.49–0.88) **P-value** 0.006
Model 3	0.09	1.10 (0.81–1.49) **P-value** 0.523	0.269	1.30 (0.96–1.78) **P-value** 0.087	-0.57	0.56 (0.41–0.77) **P-value** < 0.001	0.150	0.73 (0.54–0.98) **P-value** 0.037	0.163	0.675 (0.49–0.93) **P-value** 0.016

Model 1 without adjustment, Model 2 adjustment for HbA1c, fasting insulin, and HOMA-IR, and model 3 adjustment for the variables in model 2 and lipid profile parameters.

The analysis showed that in obese individuals, a higher level of vitamin D is associated with a lower odd of having the E3/E4 and E2/E4 genotypes compared to the E3/E3 genotype decrease by a factor of 0.6 and 0.3 respectively. Higher levels of vitamin D are linked to reduced odds of having the E3/E4 and E2/E4 genotypes in comparison to E3/E3. The association is statistically significant with a p-value of less than 0.05, even after adjusting for variables in models 2 and 3.

**Fig. 2:**
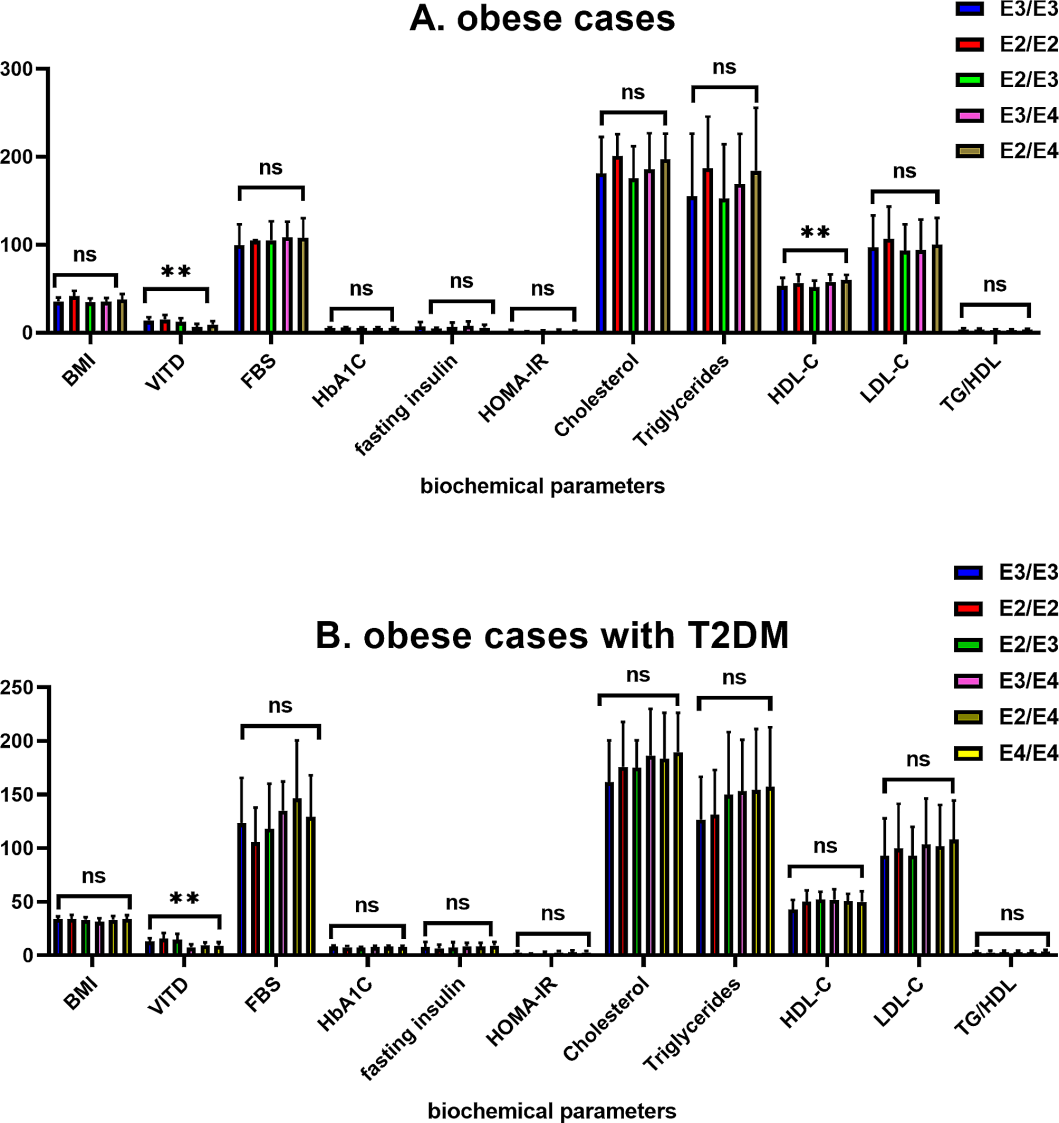
Illustrated the results of ANOVA test for biochemical parameters among studied cases, (**A**) obese cases and (**B**) obese cases with T2DM

Regarding obese individuals with T2DM, a higher level of vitamin D is associated with a lower risk of having the E3/E4, E2/E4, and E4/E4 genotypes compared to the E3/E3 genotype, which decrease by a factor of 0.5, 0.3, and 0.2, respectively. After adjusting for the variable in model 2, higher levels of vitamin D are still linked to reduced odds of having the E3/E4 and E2/E4 genotypes in comparison to E3/E3. In model 3, even with the negative correlation between vitamin D and lipid profile shown in Fig. 2, the logistic regression model shows that after adjusting for HOMA-IR and lipid profile, higher levels of vitamin D are associated with lower odds of having the E2/E4 and E4/E4 genotypes compared to E3/E3. This suggests that the effect of vitamin D on APOE genotyping is independent of its correlation with the lipid profile.

## Discussion

Recent studies have explored the link between APOE genetic variations and different diseases in Egyptians and other ethnicities. Most of them studied the correlation between APOE genotyping and T2DM, along with other conditions. Among Egyptian populations, APOE genotyping has been linked to coronary artery disease (CAD) [16, 17], coronary heart disease (CHD) [18], type 2 diabetes mellitus (T2DM) with cardiovascular disease (CVD) [19], T2DM with nephropathy (T2DMN) [20], T2DM with obesity [13], and obesity [21].

In the current study, firstly, we investigated the association between Apolipoprotein E polymorphisms and the risk of type 2 diabetes mellitus in obese Egyptian populations, and the results revealed that the Ɛ3/Ɛ4 genotype was the most frequent among all groups studied. Obese individuals with type 2 diabetes had the highest frequency of the Ɛ4 allele, whereas both the control group and obese individuals had the highest frequency of the Ɛ3 allele.

The Ɛ4/Ɛ4 genotype was associated with a higher risk of T2DM in obese cases compared to obese cases without T2DM and the control group. Conversely, it’s associated with a reduced risk of T2DM when compared to the control group alone. And the Ɛ2/Ɛ2 genotype was associated with a higher risk of both obese cases with and without T2DM compared to the control group. The E4 allele was associated with a higher risk of T2DM in obese cases compared to obese cases without T2DM and the control group, and the E2 allele was associated with a higher risk of obesity, both with and without T2DM, compared to the control group.

Various investigations have confirmed our results, indicating a substantial association between the Ɛ4/Ɛ4 genotype and Ɛ4 allele and T2DM. This association was found in research conducted in Egypt [13, 21], Saudi Arabia [22], Lebanon [23], Iran [24], Mexico [25], and Chile [26]. Several meta-analysis studies have shown that the E2 allele plays a role in the development of type 2 diabetes mellitus in various ethnic groups [27–29]. Zeljko et al. [30] found a notable association between the E2 allele and the occurrence of obesity in their study on obesity outcomes. The results were in line with our findings. Other investigations have indicated that the E4 allele plays a role in the heightened risk of obesity [12, 13, 31].

We studied Insulin Resistance and Lipid Profile and it’s associated with 25 (OH) vitamin D deficiency among studied cases, Insulin resistance, as indicated by higher levels of fasting insulin and HOMA-IR, was observed in both obese individuals and those with T2DM, compared to the control group.

The lipid profile showed significant elevations in total cholesterol, triglycerides, LDL-C levels, and TG/HDL-C ratio in obese individuals with and without T2DM compared to the control group. However, there were no significant differences between obese individuals with T2DM and the control group in certain lipid parameters.

Pearson correlation analysis revealed significant negative correlations between low vitamin D levels and fasting insulin, HOMA-IR, FBS, TC, and TG levels, particularly in obese cases and obese cases with T2DM.

Several recent research have examined the correlation between vitamin D levels, glycemic status, and lipid profile, and most of them align with our findings. Jie Zhang et al. [32], colleagues found that higher vitamin D levels may help regulate glucose balance, since 25(OH)D was inversely linked to insulin resistance in Chinese individuals with type 2 diabetes. Tran Huu el al [33], found a negative connection between 25(OH)D levels and fasting glucose level as well as HOMA-IR. In a study by Dina SM Rashad et al., pre-diabetic insulin resistant obese individuals with vitamin D deficiency experienced an improvement in weight and insulin resistance after their vitamin D levels increased [34]. A study in Turkey found a connection between vitamin D levels and insulin resistance, indicating they are not independent factors, while dyslipidemia is associated with vitamin D levels [35]. In the Saudi population, vitamin D levels were inversely connected to fasting blood glucose and hemoglobin A1c levels. Vitamin D insufficiency was closely linked to insulin resistance, particularly in obese patients, while there was no significant linkage with blood lipids [36]. The cross-sectional study used 278 young adults to investigate the correlation between 25(OH)D levels and lipid profile indicators in young individuals. The study’s results show correlations between 25(OH)D concentration and other indices of the lipid profile in blood serum. Low levels of vitamin D may be linked to a higher likelihood of dyslipidemia, particularly in males [37]. A cross-sectional research of 15,600 patients shows a substantial correlation between serum Vitamin D levels and LDL levels in patients. The results indicate that vitamin D levels may influence lipid metabolism and could be relevant for preventing and treating cardiovascular disease [38]. A hospital-based cross-sectional study conducted on Egyptian patients with multiple sclerosis. A study found that vitamin D was positively associated with HDL levels but adversely correlated with TC levels [39].

After analyzing the relationship between biochemical parameters and APOE genotyping, depicted in Fig. 1, we observed that there was a significant difference in the values of 25 (OH) vitamin D and HDL-C. However, 25 (OH) vitamin D and HDL-C demonstrated a significant difference in value based on APOE genotyping among obese cases with T2DM. Several previous investigations have indicated a contradictory connection between APOE genotyping and lipid profile compared to our findings [40–44]. Dallongeville J et al. [45] found that those with the ε4 alleles had higher plasma total cholesterol levels than those with the Ɛ3/Ɛ3 genotype. Additionally, there was a significant drop in HDL cholesterol in individuals with the Ɛ3/Ɛ4 genotype compared to those with the Ɛ3/Ɛ3 genotype. Bennet AM et al. [46] found that persons with the Ɛ2/Ɛ2 genotype had an average LDL level about 31% lower than individuals with the Ɛ4/Ɛ4 genotype. Insulin resistance closely links to metabolic dyslipidemia, and lipid profiles and diabetic phenotypes strongly interrelate. The ApoE Ɛ4 allele and its genotypes (Ɛ3/Ɛ4 and Ɛ4/Ɛ4) were linked to a higher risk of type 2 diabetes mellitus by changing the way fats are broken down [29].

And finally, by studying an association between vitamin D levels and APOE genotyping, we found that lower levels of 25 (OH) vitamin D were significantly associated with specific APOE genotypes, particularly E3/E4 and E2/E4, in both obese individuals and those with T2DM. Higher vitamin D levels in obese cases were associated with a decreased likelihood of having the E3/E4 and E2/E4 genotypes compared to the E3/E3 genotype. Also, people who were overweight and had T2DM were less likely to have the E3/E4, E2/E4, and E4/E4 genotypes if they had higher vitamin D levels compared to people who had the E3/E3 genotype.

This association remained significant even after adjusting for variables such as HbA1c, fasting insulin, HOMA-IR, and lipid profile parameters, indicating that vitamin D’s effect on APOE genotyping is independent of these factors. This negative correlation could also suggest potential pathways through which vitamin D might influence APOE genotyping, possibly through its effects on lipid metabolism.

The study suggests that vitamin D deficiency, along with APOE genotyping, may play a role in the risk of obesity and T2DM. Higher levels of vitamin D are associated with a lower risk of certain APOE genotypes, particularly in obese individuals with T2DM. These findings emphasize the importance of considering both genetic and environmental factors when understanding the pathogenesis of obesity and T2DM.

In conclusion, in obese individuals, higher levels of vitamin D were associated with a lower risk of having certain APOE genotypes compared to the E3/E3 genotype, even after adjusting for various variables. Similar associations were found in obese cases with T2DM, indicating that vitamin D levels may influence APOE genotyping independent of its correlation with lipid profile.

## Data Availability

No datasets were generated or analysed during the current study.
